# Non-invasive estimation of increased LV filling pressures in LV hypertrophy with normal systolic function: Comparison between CMR and Doppler, validated by invasive PCWP measurements

**DOI:** 10.1186/1532-429X-13-S1-O34

**Published:** 2011-02-02

**Authors:** Bernard P Paelinck, Paul L Van Herck, Johan M Bosmans, Christiaan J Vrints, Hildo J Lamb

**Affiliations:** 1 University Hospital Antwerp, Edegem, Belgium; 2 Leiden University Medical Center, Leiden, Netherlands

## Introduction

Transmitral flow is unreliable for the estimation of left ventricular (LV) filling pressures in hypertrophy and normal systolic function. Mitral early peak filling velocity E divided by mitral annulus early peak velocity Ea (E/Ea) and global diastolic strain rate (SR) during peak filling provide a relatively load independent measurement of global myocardial performance.

## Purpose

We aimed to compare Doppler and cardiovascular magnetic resonance (CMR) assessed E/Ea and non-tagged CMR assessed global diastolic SR for the estimation of filling pressure, in comparison with invasive measurement.

## Methods

Sixteen patients with hypertensive heart disease (LV mass index: 111 ± 18 g/m^2^), absence of valvular regurgitation and with normal systolic function (LV ejection fraction: 67 ± 7 %) referred for cardiac catheterization were studied. Measurement of mitral flow and mitral annulus velocities were performed by Doppler and phase-contrast CMR. CMR derived global longitudinal and global volumetric SR during early peak filling was measured using long-axis cine CMR images. These data were validated by catheter based mean pulmonary capillary wedge pressure (PCWP).

## Results

Mitral flow E/A had no significant correlation with mean PCWP. E/Ea (Doppler r= 0.74, p<0.01 and CMR r=0.56, p<0.05), longitudinal diastolic SR (r=0.65, p<0.01) and long-axis volumetric diastolic SR (r=0.51, p<0.05) related to invasively measured mean PCWP.

Best prediction of elevated mean PCWP was performed by Doppler assessed E/Ea (sensitivity 75%, specificity: 100%, area under the curve: 0.88, p<0,05). CMR assessed E/Ea, longitudinal volumetric diastolic SR and long-axis volumetric diastolic SR had similar sensitivity (respectively 75%, 87.5 % and 75 %), specificity (respectively 75%, 62.5 % and 87.5 %) and area under the curve (respectively 0.80, 0.80 and 0.80, p<0.05) for the prediction of elevated mean PCWP.

## Conclusions

Non-invasive estimation of increased LV filling pressures in LV hypertrophy with normal systolic function can be performed with CMR and Doppler techniques. Doppler assessed E/Ea provided best prediction of elevated LV filling pressure.

